# Problems with the concept of gut microbiota dysbiosis

**DOI:** 10.1111/1751-7915.13479

**Published:** 2019-08-26

**Authors:** Harald Brüssow

**Affiliations:** ^1^ Laboratory of Gene Technology Department of Biosystems KU Leuven Leuven Belgium

## Abstract

The human microbiome research is with the notable exception of fecal transplantation still mostly in a descriptive phase. Part of the difficulty for translating research into medical interventions is due to the large compositional complexity of the microbiome resulting in datasets that need sophisticated statistical methods for their analysis and do not lend to industrial applications. Another part of the difficulty might be due to logical flaws in terminology particularly concerning ‘dysbiosis’ that avoids circular conclusions and is based on sound ecological and evolutionary reasoning. Many case–control studies are underpowered necessitating more meta‐analyses that sort out consistent from spurious dysbiosis–disease associations. We also need for the microbiome a transition from statistical associations to causal relationships with diseases that fulfil a set of modified Koch's postulates for commensals. Disturbingly, the most sophisticated statistical analyses explain only a small percentage of the variance in the microbiome. Microbe–microbe interactions irrelevant to the host and stochastic processes might play a greater role than anticipated. To satisfy the concept of Karl Popper about conjectures and refutations in the scientific process, we should also conduct more experiments that try to refute the role of the commensal gut microbiota for human health and disease.

## Graphical abstract

Microbiome research has with the exception of fecal transplantation not yet reached the level of industrial applications with respect to gut microbiome engineering. Part of the difficulty might be the\complexity of the system, but some problems might reflect imprecise definitions like that for dysbiosis. Refutations of concepts should be part of the scientific process.

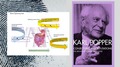

## Introduction

‘The human microbiota is the focus of one of the most dynamic research fields of our time’ – with this sentence starts a recent Cell review on the human gut microbiome (Schmidt *et al.,* 2018). When looking to the coverage of microbiome research in Nature, Science, Cell and their sister journals, this judgement is by no means an exaggeration. Sometimes entire issues are packed with microbiome papers, like the 30 May 2019 issue of Nature presenting the latest results from the Integrative Human Microbiome Project (HMP) (Proctor and the Integrative HMP (iHMP) Research Network Consortium, 2019.

One of the three subprojects of HMP deals with the gut microbiota in inflammatory bowel disease (IBD), where an impressive array of multiomics technologies have been applied to nearly 3000 stool, biopsy and blood samples taken repetitively over one year from 132 patients (Lloyd‐Price *et al*., 2019). Interindividual variation accounted for the majority of the observed variance, while disease status explained only a smaller proportion. During the follow‐up, ‘dysbiotic’ gut microbiome excursions occurred in some patients, but were only weakly associated with disease activity. These gut microbiota deviations thus represented potentially stochastic events. Taxonomic shifts to aerotolerant, proinflammatory bacterial clades confirmed observations from previous reports. New are observations of greater gene expression by clostridia and a reduction in an unclassified *Subdoligranulum* species in IBD. The authors stressed that it is unclear whether multiomics microbiome data can predict disease events before their occurrence. Causal analysis needs intervention study designs. The data provide a catalogue of new relationships between multiomics features that enable future research, yet microbiome research remains thus largely descriptive.

According to the coordinator of this project, progress in microbiome research has excited industry (Proctor and the Integrative HMP (iHMP) Research Network Consortium, 2019; Proctor, 2019). Financial figures are useful to put the microbiome enterprise into perspective for the microbial biotechnologist. Over the last decade, more than US$ 1.7 billion in research money has been spent on microbiome projects by the public sector. The market value of microbiome‐based products and interventions (diagnostic and therapeutic) is currently estimated to be between US$ 275 and 400 million worldwide and is expected to at least triple over the next years (Proctor and the Integrative HMP (iHMP) Research Network Consortium, 2019; Proctor, 2019). In comparison, the global market for probiotics amounted to US$ 40 billion in 2017 (Reid *et al*., 2019). Apparently, there are difficulties in translating basic microbiome research into food, nutrition and health or pharmaceutical products. One notable exception is faecal transplantation for the treatment of recurrent *Clostridium difficile* infections (CDI). However, despite clear data on its efficacy (Tariq *et al*., 2019), its development into a commercial product meets regulatory hurdles (Vyas *et al*., 2015; Verbeke *et al*., 2017), and the FDA has recently issued a safety alert pertaining to its use (https://www.fda.gov/vaccines-blood-biologics/safety-availability-biologics/important-safety-alert-regarding-use-fecal-microbiota-transplantation-and-risk-serious-adverse). Furthermore, the basic concept of this approach predates microbiome research (de Vos, 2013), and defined bacterial strains for recurrent CDI (Tvede and Rask‐Madsen, 1989; Tvede *et al*., 2015) still need to be developed into registered products.

### The problem of definitions

The problems confronting microbiome‐derived products for industrial applications are manifold. It might surprise that one hurdle is a question of basic scientific definition, an issue that should not be dismissed as of purely semantic importance. Nearly 2000 years ago, a founding father of medicine stressed the importance of clear scientific terms when writing ‘First, however, we must distinguish and explain clearly the various terms which we are going to use and to what things we apply them; and this will prove not merely an explanation of terms but at the same time a demonstration of the effects of nature’ (Galen, 191 AD). The probiotic field has long discussed what represents a probiotic, and consensus conferences have been organized to find a definition. Having a clear definition does not alone solve scientific questions. In fact, learned societies have only recommended few probiotics for a limited number of health applications (Brüssow, 2019). However, lacking a clear definition of terms risks blurring the discussion.

The microbiome field employs the overarching term ‘dysbiosis’ and its correction to a state of ‘eubiosis’ by targeted interventions. The difference to the probiotic field is that no consensus conference has yet worked out a definition of ‘dysbiosis’ and many microbiome research papers use this term without even an *ad hoc* definition or specification. This neglect illustrates not only a lack of scientific rigour, but might in fact hamper progress in the microbiome field from a descriptive into a translational science. Over recent years, criticism of the undefined use of the ‘dysbiosis’ term has been repetitively articulated. In a short comment, ‘Dysbiosis is not an answer’, Olesen and Alm (2016) dismissed dysbiosis as a major organizing concept in microbiome science, arguing that it is based on pre‐scientific thoughts of microbial imbalances and resembling somewhat the humoural theory of human health. According to these authors, the ambiguity of the definition allows to observe dysbiosis in microbiota composition without actually accomplishing anything scientifically useful. They quote diagnostic microbiome signatures for inflammatory bowel disease (IBD) as an example, which are not superior to the simpler test based on faecal calprotectin measurement. In addition, diagnosing dysbiosis does not tell whether it is a cause or an effect of disease. A detailed historical evaluation of the term dysbiosis and its current use and misuse is provided by Hooks and O'Malley (2017). When they screened microbiota literature, they found ‘imbalance of the microbiota’ as the most common characterization of dysbiosis, with imbalance defined as a loss of homoeostasis, which itself is not, or only rarely, defined, therefore leading to a circular definition in most applications. This vague definition led, in the view of these authors, to a catchall definition lacking scientific value, which has hampered the microbiome field to progress from an association‐focused to an explanatory science.

### Approaches to defining dysbiosis

Levy *et al*. (2017) distinguished three types of dysbiosis defined as the ‘bloom of pathobionts, loss of commensals, or loss of diversity’, while Vangay *et al*. (2015) distinguished four dysbiosis types, namely ‘loss of keystone taxa, loss of diversity, shifts in metabolic capacity, or blooms of pathogens’. Pathobionts are defined here as members of the commensal microbiota that have the potential to cause pathology (Chow and Mazmanian, 2010). A keystone species is defined by having a disproportionally large effect on its natural environment relative to its abundance. Many definitions tried to link dysbiosis with disease such as ‘dysbiosis is any change in composition of resident commensal communities relative to the community found in healthy individuals’ (Petersen and Round, 2014). According to Hooks and O'Malley (2017), this definition pointed to a major methodological problem of the field: by searching for microbiota changes in ill people compared with healthy subjects in case–control studies, the dysbiotic state is tacitly confirmed as conferring illness, which is a classical circular conclusion. Or in a milder form of criticism, Bäckhed *et al*. (2012) stated that ‘current evidence is insufficient to distinguish between dysbiosis as a cause or consequence of the disease’.

Fundamental criticism on the naïve use of the term dysbiosis was formulated by Levy *et al*. (2017), who observed that it is not sufficient to compare diseased individuals with a disease‐free control cohort; it particularly needs comparisons across different manifestations of the same disease. This request would provide a closer level of association without, however, entirely solving the cause or consequence problem. These authors also called for functional rather than taxonomical interpretations of the dysbiotic microbiota and raised the point of a high contextual dependence of observations, for example with the host immune system acting on the microbiome and the microbiome acting on the host immune system. These authors come with a radical proposal that the definition of a dysbiotic microbiota configuration should fulfil criteria of Koch's postulates for the definition of a disease‐causing microbial agent, here extended for a disease‐causing microbiome.

When investigating the link between paediatric dysbiosis and disease, Vangay *et al*. (2015) mentioned important confounding factors that need to be accounted for in such microbiota–disease analyses, like the temporal maturation of the gut microbiota, and the dependence of the microbiota composition on the type of delivery (Caesarian vs. vaginal) (Chu *et al.*, 2017), on diet (breast‐ vs. bottle‐feeding) and on prior antibiotic use. A detailed description of the gut microbiota from infants taking account of these factors has been provided for healthy infants from Sweden (Bäckhed *et al*., 2015) and the United States (Baumann‐Dudenhoeffer *et al*., 2018). A single case–control study has documented a microbiota–disease association, namely malnutrition in children from Bangladesh, with the microbiota age development by using machine learning on 16S RNA sequence data that resulted in a ‘relative microbiota maturity index’. The authors found a relative microbiota immaturity for malnourished children (Subramanian *et al*., 2014). While this observation is of research interest, it is of limited practical importance since this condition can be easily assessed by anthropometry and visual diagnosis.

### The issue of diversity

Loss of diversity has been invoked as a criterion for dysbiosis in many definitions, suggesting a decreased microbial service to the host and thus of lesser microbiota ‘health’. However, authors considering the human gut microbiota from the viewpoint of current ecological theory came to more nuanced evaluations (Coyte *et al*., 2015). High species diversity is undeniably a characteristic of the gut microbiota. While increased cooperation within such a complex community is expected to promote overall metabolic efficacy, the host faces a trade‐off since it comes at the cost of decreased ecological stability because high species numbers tend to destabilize the system. Johnson and Burnet (2016) therefore called for caution towards a naïve application of the diversity argument. For example, bottle‐fed babies consistently showed a more diverse gut microbiota than breastfed infants (see Simeoni *et al*., 2016, as an example) without this difference translating into a greater health of bottle‐fed over breastfed infants. Another example is frequently quoted in this context: an African hunter‐gatherer population (the Hadza) showed greater taxonomical (Schnorr *et al*., 2014) and functional diversity (Smits *et al*., 2017) of the gut microbiota than subjects from industrialized countries. To infer that the Hadza gut microbiota constellation is associated with a better ‘gut health’ than that of people from industrialized countries on the diversity argument without epidemiological data or health examination would be more or less a mere postulation. This conclusion is based on theoretical concepts like that of a mutualistic superorganism, where microbes are qualified as ‘old friends’ that have co‐evolved during hominid evolution and declined with adaptation to more modern food production systems. However, as long as these data are not backed by actual health metrics, they should be interpreted with caution. Actually, the Hadzas lacked bifidobacteria (Schnorr *et al*., 2014), which are generally considered to confer health effects to the gut microbiota and which are a common heritage of hominid evolution (Moeller *et al.,* 2016). If the lack of bifidobacteria is not a technical artefact (e.g. question of DNA extraction, primer use as in several studies failing to report bifidobacteria), it might indeed suggest an unhealthy gut microbiota, especially in addition to the observation of Treponema in the stool of the Hadzas, which are generally considered as unhealthy. Indeed, six per cent of the Hadzas showed Treponemas also in the serum samples, raising further doubts about their overall health status. While we have data that show how microbiota composition changes occur in a genetically homogenous population transitioning to different food production systems, we still lack data on how these changes affect human physiology and health along this gradient (Jha *et al*., 2018).

The Hadza data underline another aspect, namely the importance of seasonal variability of the gut microbiota in primitive societies (Smits *et al*., 2017), which compromises hopes to associate gut microbiota constellation with health unless also anticipating seasonal health changes or two different healthy gut microbiota constellations in different ecological settings. There are even data, although elaborated with very small numbers of human subjects, demonstrating diurnal changes in gut microbiota composition and described with more detail in mice (Thaiss *et al*., 2014). If confirmed, such volatility of the gut microbiota constellation would necessitate protocols with standardized stool sampling times (seasons, hours of the day) to support comparisons across studies and allow generalizations to be deduced.

Also, Shade (2017) calls for restraint in using diversity measurement for health assessments: diversity alone has limited value because much context is needed for its interpretation. Discrepancy between different indices of community diversity has long been recognized in the field of ecology, thus complicating comparisons across studies. As such, microbiota diversity is neither good nor bad. The point is easily demonstrated by the human vaginal microbiota, where increased microbial diversity is clearly associated with detrimental effects (bacterial vaginosis, in Afro‐American women even associated with compromised birth outcomes; Fettweis *et al*., 2019). The host genetic background dependency for microbiota–health associations is well demonstrated in vaginal microbiota studies from Afro‐American and White US women (Callahan *et al*., 2017). In the case of the vaginal microbiota, the argument of a co‐evolution of a microbiota with the hominid lineage is not applicable since a *Lactobacillus*‐dominated vaginal microbiota is only found in humans and not in apes (Miller *et al*., 2016).

### Underpowered studies and meta‐analyses

Many microbiota studies diagnosing dysbiosis in case–control studies are statistically underpowered, counting only small human sample sizes: < 20 subjects per group is not unusual in the field. To account for these limitations, Duvallet (2018) argue for meta‐analyses in the dysbiosis field. A meta‐analysis can increase the statistical power of many small studies by detecting true signals against a background of false positives. A major asset of meta‐analysis is the identification of consistent observations across independent studies or the refutation of spurious associations. A test case is provided for the gut microbiota association with obesity.

Initial studies had reported differences in gut microbial community diversity and differences in the Bacteroidetes/Firmicutes (B/F) ratio among obese and non‐obese individuals. A pioneer study was done with small subject numbers (12 obese and 5 lean subjects), showing that the B/F ratio increased with time on a calorie‐restricted diet (Ley *et al*., 2006). A follow‐up study in twins enrolling 154 individuals confirmed that obesity is associated with phylum‐level changes in the gut microbiota (decreases in Bacteroidetes and increases in Actinobacteria abundance), reduced bacterial diversity and altered bacterial gene expression (Turnbaugh *et al*., 2009). Together with similar microbiota changes observable in obese and lean mice, the demonstration that the ‘obese’ microbiota had an increased capacity to extract energy from the diet and the demonstration that this trait is transmissible to germ‐free mice (Turnbaugh *et al*., 2006), the obesity–microbiota connection became a showcase for pronounced division‐wide changes in microbial gut ecology associated with host pathology. However, a meta‐analysis including 10 case–control studies and approximately 2800 subjects (about a third of whom were obese) did not reveal a significant difference in B/F ratio between lean and obese groups and only marginal differences for microbiota diversity (Sze and Schloss, 2016). Finucane *et al*. (2014) used database mining in the US Human Microbiome Project (HMP) and the European MetaHIT data set and found no associations between B/F ratio and body mass index (BMI) or phylum‐level microbiome composition and BMI. The between‐study variability in the relative abundance of Bacteroidetes and Firmicutes was greater in their analysis than the within‐study differences between lean and obese individuals. In contrast, Falony *et al*. (2016) found an association, albeit a small one, between microbiome composition and BMI when analysing the gut microbiome in 1106 subjects from the Flemish Gut Flora Project (FGFP). However, based on their data, they estimated that one would need 865 lean and 865 obese volunteers to detect microbiota compositional shifts with a *P* < 5% significance level and a power of 80%. No single obesity study of this size has yet been conducted.

It would certainly be desirable that future microbiome–disease association studies conduct power calculations with realistically anticipated effect sizes in their published results, as routinely done in the protocols for clinical trials. Such approaches plus meta‐analyses will help to distinguish true from spurious associations to allow focusing on promising microbiota–disease associations that lead the field from hype to hope (McKenney & Pamer 2015), with meta‐analyses of faecal transfer in *C. difficile* infection serving as an example (Tariq *et al*., 2019).

### Categorizations of dysbiosis studies

Gilbert *et al*. (2016) distinguished three categories of microbiota–disease associations: predictable associations (e.g. irritable bowel syndrome, inflammatory bowel syndrome), intriguing associations (e.g. obesity, cardiovascular disease, colon cancer, rheumatoid arthritis) and surprising associations (e.g. major depression, Parkinson disease, autism), which might also represent gradients of likelihood for microbiota being causally involved in diseases or the time needed for translation of research data into clinical practice. Duvallet *et al*. (2017) used a meta‐analysis approach of case–control studies across different disease types that led to interesting conclusions. They attributed microbiota changes into distinct categories. One category is characterized by an enrichment of a small number of potential pathogens like *Fusobacterium, Porphyromonas, Peptostreptococcus, Parvimonas* and *Enterobacter*, as identified in three of four colorectal cancer studies (Wang *et al*., 2011; Chen *et al*., 2012; Zeller *et al*., 2014; Baxter *et al*., 2016). These studies suggest specific antimicrobials as therapeutic approaches (e.g. bacteriocins or bacteriophages). Another category corresponded to diseases associated with a depletion of health‐associated bacteria. For example, five genera from the *Ruminococcaceae* and *Lachnospiraceae* families were consistently depleted in IBD patients. These studies suggest probiotic approaches in order to supplement the missing bacteria from the community. A few diseases were characterized by gross changes of microbiota composition, with *C. difficile* diarrhoea being the clearest example, suggesting faecal transplantation as verified by multiple clinical studies. Some microbiota–disease associations were likely due to confounding factors, such as HIV‐associated microbiota changes influenced by sex practices or obesity–microbiota associations influenced by diet effects. A few bacteria were apparently non‐specifically associated with several diseases: *Escherichia/Shigella/Salmonella* induced by antibiotic treatments independent of the underlying infection type.

### Eubiosis

The relatively elusive character of the term ‘dysbiosis’ is the mirror image of the likewise poorly characterized opposite term of ‘eubiosis’. No precise definition has been given for eubiosis beyond a ‘balanced’ microbiota or a microbiota found in healthy subjects. The clear definition of the eubiotic microbiota is particularly important for case–control studies. If the healthy eubiotic microbiota is well defined, both semantically and by its microbiota composition, only microbiota data from a small numbers of control subjects would be needed. This effect is proposed by the ‘Anna Karenina principle’, which states that dysbiotic individuals vary more in microbial community composition than healthy individuals (Zaneveld *et al*., 2017). The peculiar name of this principle is a pun on a book of Leo Tolstoy, which starts with the sentence: ‘All happy families look alike, each unhappy family is unhappy in its own way’. Support for this principle was found in ocean corals where dysbiotic corals, i.e. stressed corals, have a more variable and unstable microbiome than healthy ones (Ahmed *et al*., 2019). While the human vaginal microbiota seems to concur with this principle, it is far from clear whether this principle applies to the healthy human gut microbiota (Brüssow, 2016).

If the gut microbiota composition in healthy subjects is highly variable, large numbers of subjects are needed in case–control studies to arrive at significant conclusions. An estimate for the numbers needed can be derived from large microbiome studies of average health individuals from Belgium (Falony *et al.,* 2016) or The Netherlands (Zhernakova *et al.,* 2016). A microbial census yielded about 800 bacterial genera. Yet, the richness of western gut microbiota is still undersampled and the authors estimated that it would need 40 000 subjects to arrive at saturation. The Belgian study yielded 20 core taxa (present in 95% of the samples), and core taxa also belonged to the most abundant taxa. Variation between individuals was in fact substantial, but resulted mainly from changes in the abundance of core taxa like *Ruminococcaceae, Bacteroides* and *Prevotella*, all organisms that had previously been proposed as enterotype identifiers (Arumugam *et al.,* 2011). In addition to these three major groups of gut bacteria, a long tail of low abundance bacteria was described that contributed substantially to the functional diversification of the healthy gut microbiota (Arumugam *et al.,* 2011). About 70 factors from the subjects showed significant associations with the microbiota composition, and nearly half of them were also identified in the Dutch cohort as significant covariables (Falony *et al.,* 2016). However, these factors explained a mere 1–15% of the genus abundance variation, therefore suggesting that unknown effects, biotic interactions and even stochastic processes have major influences on the healthy gut microbiota. A strong stochastic element was also manifest in substantial week‐to‐week gut microbiota changes in healthy children (Sarker *et al*., 2017a) and day‐to‐day variation in healthy adults from Bangladesh (Sarker *et al*., 2012; McCallin *et al*., 2013). If confirmed by studies from other geographical areas (the high variability of gut microbiota in Bangladesh might reflect a low environmental and food hygiene level), it will be difficult to differentiate dysbiotic microbiome markers from small numbers of controls, particularly if it affects abundance changes in major taxa like the B/F ratio that showed a continuum of variation also in European subjects, as demonstrated by an elderly study from Ireland (Claesson *et al*., 2011).

### Confounding factors

Medication for everyday life conditions had the greatest impact on microbiota composition in the Belgian FGFP and the Dutch LifeLines DEEP studies (Falony *et al.,* 2016; Zhernakova *et al.,* 2016). This observation is not surprising in view of the impact of non‐antibiotic drugs on commensal bacteria: 24% of 1000 common drugs inhibited bacterial growth *in vitro* (Maier *et al*., 2018). The strong impact of medication as a confounding factor is shown in studies investigating the association of gut microbiota dysbiosis with type 2 diabetes (T2D). A Chinese study described a moderate gut microbiota dysbiosis in a T2D case–control study, where only 4% of the gut microbial genes were associated with T2D. Functional annotation indicated a decrease in butyrate‐producing bacteria and an increase in facultative pathogens (Qin *et al.,* 2012). A Danish study associated increases in four *Lactobacillus* species and decreases in five *Clostridium* species in women with T2D. Microbial classifiers (*Lactobacillus, Akkermansia*) differed between the Chinese and Danish T2D cohorts, pointing to population‐specific effects (Karlsson *et al.,* 2013). A third study stratified T2D patients according to medication with metformin (Forslund *et al.,* 2015). In metformin‐naïve patients, a decrease in butyrate‐producers was associated with an increase in *Lactobacillus* with disease. However, in metformin‐treated T2D patients, the same authors associated a significant increase of *Escherichia* with disease, which might explain both the therapeutic and adverse effects (diarrhoea, bloating) of this most widely used antidiabetic medication. Case–control studies must therefore be stratified for medication in order to provide reliable microbiota–disease associations. Otherwise, microbiota changes could simply be a consequence of disease treatment.

### Multiple omics approaches…

Due to the substantial interindividual variability and the influence of confounding factors on the gut microbiota, other authors have explored more sophisticated approaches to differentiate healthy from dysbiotic microbiota. One option is to explore the temporal variability with mathematical approaches from theoretical ecology. The two‐dimensional parameter space of Taylor's law allowed a definition of a healthy microbiota space from which antibiotic‐treated subjects, morbid obese patients and irritable bowel syndrome (IBS) patients could be differentiated (Marti *et al.,* 2017). Gilbert *et al*. (2016) recommend time‐series studies for microbiome‐wide association studies to link microbiota changes to disease. They argue for the establishment of a type of microbial Global Positioning System (GPS), integrating microbiome, host genome and disease subtype differentiation data obtained with multiple omics approaches. In this way, they suggest to follow the microbiome path of at‐risk subjects and patients through a principal coordinate analysis (PCoA) plot. Such plots could be of diagnostic use when identifying patients at risk before a disease becomes manifest and would also allow to follow the impact of therapeutic interventions by changes in plot positions. In such a plot analysis, diet interventions considered as mild are supposed to confer small shifts in plot position, while strong (e.g. antibiotics) interventions induce large shifts and game‐changing interventions (e.g. faecal transplantation) would lead to ‘teleportation’ of the patient's microbiota to the healthy area in these plots.

The trend in analysing the healthy gut microbiome and its dysbiotic changes is clearly towards using tools of increasing analytical complexity. One of the latest developments is borrowed from statistical approaches developed to deal with interacting systems of seemingly intractable complexity that was initially applied to the analysis of financial markets (Raman *et al*., 2019). A large international consortium led by J.I. Gordon, a pioneer of microbiota–obesity and microbiota–malnutrition studies, conducted statistical covariance analysis with stool samples from a Bangladeshi birth cohort, which revealed an ‘ecogroup’ of 15 co‐varying bacterial taxa that provide a concise description of the microbiota development in healthy children that was also applicable for children from India and Peru. The primary principal component described 80% of the data variance. The ecogroup analysis allowed a clear differentiation of healthy children from severely malnourished children (SAM) and a weaker differentiation of children with moderate malnutrition (MAM) from healthy controls. Conventional re‐feeding therapy caused marked shifts in the principal component analysis (PCA) space of microbiota composition without reaching the PCA position of healthy controls. Aspects of microbe–microbe interaction could be reproduced in a gnotobiotic piglet model.

### …and their limitations

Approaches that create a microbial GPS are fascinating, but still far away from translation into clinical practice. The sensitivity and specificity of microbiota tests for disease diagnosis are still relatively unclear. It is also unknown whether microbiota changes are sufficiently fine‐grained to allow diagnostically meaningful disease differentiation. More fundamentally, the majority of the gut microbiota studies have been conducted for logistic reasons with stool samples. The microbiota composition of faecal samples differs from that of mucosal samples (Eckburg *et al*., 2005). Obviously, the mucosal microbiota, due to its closer association with the diseased gut tissues, is more likely to affect disease outcome if the microbiota are drivers of the investigated disease. The faecal microbiota in contrast is a mixture of shed mucosal bacteria and a separate non‐adherent luminal microbiota, which is less likely to reflect the disease process. The faecal microbiota is thus potentially only a distant mirror of pathological events, and therefore, we should not expect close correlations with gut disease even if they existed for the mucosal microbiota.

### A comprehensive, complex intervention trial

The Gordon‐led consortium extended their ecotype analysis (Raman *et al*., 2019) into a nutritional intervention trial to support the hypothesis that a healthy microbiota development is causally linked to healthy growth (Gehrig *et al*., 2019). As formulated, this sounds a bit like a circular conclusion. Their starting observation is that malnourished children from Bangladesh showed a delayed maturation of their gut microbiota compared with control children (Subramanian *et al*., 2014). They designed nutritional components from Bangladesh from microbial inoculation experiments in gnotobiotic mice and piglets that affected a shift from a malnourished to a healthy gut microbiota associated with biomarkers of health and growth in experimental animals. Based on this pre‐selection, they conducted a randomized, double‐blinded trial with four different feeding regimes in moderately malnourished MAM children (14‐17 children per group). The intervention led to a statistically significant, but clinically small, weight increase (weight‐per‐height Z‐scores ameliorated from −2.2 to −1.9) in all four groups. The microbiota composition of the MAM children, which was already close to that of healthy controls, showed a shift towards the healthy gut microbiota composition for the microbiota‐directed complementary food 2 (MDCF2) group, who were fed with chickpea, soya and peanut flour plus bananas. In parallel, the authors observed a change to a ‘healthy growth discriminatory’ plasma proteome derived from a comparison of SAM, MAM and healthy children. The data should be interpreted with caution since the intervention was conducted in children with moderate malnutrition who showed already a gut microbiota composition close to healthy children at enrolment. Furthermore, the weight gaining effect was modest and the end‐points were biomarkers and not a clinical assessment. One of the significant alterations affected by MDCF2 was a decrease in *Bifidobacterium longum*. This conclusion is counterintuitive since bifidobacteria are associated with breastfeeding (Simeoni *et al*., 2016), commonly regarded as desirable infant nutrition, and bifidobacteria are leading probiotic candidate in paediatrics (Brüssow, 2019) and health‐associated gut species in elderlies (Brüssow, 2013). It remains to be seen whether this microbiota‐designed nutritional intervention will have the desired growth effects in future clinical trials. The causal link between this dysbiotic gut microbiota and malnutrition, while perhaps suggestive, is not proven. Proof might need an approach designed according to Koch's postulates for disease‐associated pathogens.

### Modified Koch's postulate for microbiome–health associations

Researchers from the Sanger Institute have suggested postulates for health‐associated microbial commensals (Neville *et al*., 2018). Their first postulate requires that the health‐associated commensal is regularly identified in healthy hosts and less frequently in disease hosts. This sounds much like the commonly applied dysbiosis criterion of case–control studies, but differs in important points. Since the human microbiota is highly variable, the authors request large data sets to identify robust signals for fulfilment of this postulate. Furthermore, high‐resolution identification at the strain level is needed for this first postulate. Identification at a species level, not to speak of genus or even higher taxonomical levels, is not sufficient. Fulfilment of their second postulate requires pure culture isolation of the identified commensal. Such a request sounded unrealistic. However, the isolation of more than 1000 taxonomically identified bacterial strains from a single human adult stool by the limiting dilution technique (Goodman *et al*., 2011) demonstrated that such an approach is feasible, albeit labour‐intensive. The Sanger scientists demonstrated that 234 isolates representing 134 species and corresponding to 90% of the abundance of stool bacteria at species level could be cultivated even when using a single medium (Browne *et al*., 2016). Their third postulate stipulates that the commensal strain(s) ameliorates disease when introduced into a new host. The *in vivo* model should be a biologically relevant vertebrate model. Mice are commonly used for this demonstration, where mixtures of commensals (Lawley *et al*., 2012) and even single commensal strains (Buffie *et al*., 2015) showed prophylactic and therapeutic activity against *C. difficile* or *Salmonella enterica* infections (Brugiroux *et al*., 2016). As for the original Koch's third postulate, this criterion is a work‐intensive, but necessary approach that is complicated by biological intricacies of the mouse model that might limit extension to the human condition and thus translational research (Arrieta *et al*., 2016). The fourth postulate requires that the commensal strain can be detected following its introduction into a host who experienced health amelioration. Since this detection can be done by PCR, this criterion seems highly feasible, but caveats exist because commensals might not need to persist in order to mediate a therapeutic effect if they downregulate inflammatory processes or increase gut barrier functions and become thereafter dispensable (Li *et al*., 2016), as postulated for some probiotics.

When applying Koch‐type postulates for the commensal–health, and by extension to the dysbiosis–disease, connection, one realizes how far we are still from a clear definition of the field, beyond the possible exception of commensals and *C. difficile* infection. However, in that area of clear clinical benefit by faecal transplantation, we are, except for an approach predating the microbiome era (Tvede and Rask‐Madsen, 1989; Tvede *et al*., 2015), not yet at an intervention level with microbial elements defined at the strain level.

### On conjectures and refutations

The concept of eubiosis is linked to another concept, that of a holobiont or a hologenome (the collective genome of host and microbiome). Proponents of this concept state that all animals and plants establish symbiotic relationships with microorganisms, which are transmitted between generations and affect the fitness of the holobiont within its environment, thus leading to a type of superorganism and a new unit of selection in evolution (Zilber‐Rosenberg and Rosenberg, 2008). This concept seems to influence many microbiologists with the tacit assumption that a disturbance on the microbiome side (dysbiosis) should lead to fitness loss of the holobiont and to health disturbances in pronounced cases in the animal host. Evolutionary biologists are rather sceptical about this concept, not least because it introduces Lamarckian elements into Darwinian evolution. Moran and Sloan (2015) argued that the hypothesis stating that host‐specific microbial community compositions have evolved for the benefit of the host should not be accepted as the null hypothesis for explaining features of host–symbiont associations. While this can be the case, for example, in corals (which show, however, a much more intimate animal–microbe relationship), this case cannot be generalized. Conflicts between hosts and their associated microbes are common even with the closest imaginable host–microbe (mitochondria) interaction resulting in cytonuclear conflicts. Many more situations in the gut will, in addition, reflect microbe–microbe conflicts without effects on the host. These unseen intermicrobial conflicts might suggest a high influence of stochastic processes in human gut microbiome compositions (Falony *et al.,* 2016, Zhernakova *et al.,* 2016). Instead of anticipating without further proof the detrimental effects of microbial dysbiosis on human health while they reflect only inner‐microbial competition, we should look for cases that fulfil the modified Koch's postulates for microbial commensals that mediate human health. Otherwise, the dysbiosis – like the holobiont – concept risks to cause more confusion than clarity. The philosopher Popper (2002) has stated that knowledge acquisition, and thus also scientific research, consists of the dialectic process of conjectures on the one side and refutations on the other side. Conjectures are manifold in the microbiome field, giving the impression that we are at the threshold of a ‘New Biology’. Instead of creating more and more thrilling conjectures in that field, not only should we look for putting them on a firm conceptional ground with modified Koch‐like postulates, we should also actively seek refutations of the microbiome working hypotheses. The refutation arm of knowledge building is currently underrated in the scientific community. High‐impact journals and grant agencies look more for stimulating new conjectures than for down to the earth refutations of them. However, this unequal rating of the two arms of knowledge building leads to a serious underuse of the refutation arm for the advancement of knowledge.

### Experiences with diarrhoea

Instead of ending with this rather theoretical outlook, I will shortly mention our own experiences in the dysbiosis–health and disease field. When we tried to treat children suffering from *E. coli* diarrhoea with coliphages (phage therapy) in Bangladesh, we realized that the faecal microbiome of the patients displayed a marked dysbiosis with increased streptococcal abundance compared with local healthy control children (Sarker *et al*., 2016). *E. coli* titres were not prominent and did not correlate with quantitative diarrhoea parameters, while stool streptococci did. With recovery from diarrhoea, the streptococcal abundance decreased and the faecal microbiota approached that of control children. It was thus tempting to associate the faecal streptococcal abundance increase with diarrhoea. However, the streptococci belonged to two commensal groups (*S. salivarius* and *S. bovis* species complex) and genome sequences from stool isolates showed no virulence factors (Sarker *et al*., 2016). In subsequent studies, we found the same faecal streptococcal abundance increase in diarrhoea patients irrespective of diarrhoea aetiology, even including patients with rotavirus, a clearly defined paediatric diarrhoea pathogen (Kieser *et al*., 2018). When correcting for stool bacterial counts and stool volumes, quantitatively determined streptococcal stool output was only weakly increased compared with control children. An increase in relative abundance should always be corrected for total bacterial counts: a trivial, but essential control (Vandeputte *et al*., 2017) neglected in many microbiome studies. The streptococcal abundance increase and its apparent association with diarrhoea might simply be a consequence of the elimination of the typical colon microbiota by the watery diarrhoea pathology (purging), leading to a relative prominence of faecal streptococci, which are indeed commensals of the small intestine and perhaps less affected by gut emptying since the peristaltic flow in the small intestine is anyways high (Brüssow, 2016). In two other special groups of diarrhoea patients (malnourished children with acute diarrhoea, children with persistent diarrhoea), we observed another faecal microbiota dysbiosis (Kieser *et al*., 2017, Sultana, submitted). This time, we observed an *E. coli* increase documented with both abundance increase and absolute titre increase compared with control children. While *E. coli* could be a pathogen for these forms of diarrhoea (Sarker *et al*., 2017b), metagenome sequencing did not suggest diarrhoea‐specific virulence gene increases in the stools of patients. Based on clinical data and evidence from mouse models (Faber *et al*., 2016), it seems more likely that their increase is a consequence of treatment with antibiotics. In all of these cases, the diarrhoea–dysbiosis association is more likely to represent a consequence rather than a cause for diarrhoea. Also, the observation that the dysbiosis ameliorates with recovery from diarrhoea is not a strong argument for a causal association.

Acute diarrhoea is an interesting test case for microbiome research since the disease is of short duration and self‐limiting. It represents a natural perturbation of the physiological gut microbiota equilibrium and its disturbance by the pathological process and the re‐establishment of a new equilibrium holds promises to understand the mechanisms of microbe–microbe interaction in the gut.

### Outlook

Medical interventions, like antibiotic treatment or gut cleansing in preparation for colonoscopy represent interesting research opportunities for microbiome analyses since all enrolled subjects are informative and biological samples can be easily obtained before, during and after interventions (Fukuyama *et al*., 2017). These data can complement the insights from acute diarrhoea studies.

A critical test for a causative effect of dysbiosis on disease can only be provided by prospective studies where microbiota composition is regularly established for all participants before diseases are occurring. Such approaches are labour‐intensive and costly, even for diseases occurring with high frequency, like diarrhoea in children from developing countries. If the suspected disease‐causing dysbiosis is then observed in subjects before they develop the manifest disease, but not in age‐ and milieu‐matched children, who do not develop the specified disease, a causative role can be reasonably anticipated. A definitive proof will still need evidence for a mechanism of action by the dysbiosis. We are currently conducting a birth cohort study in Bangladesh with nearly 300 children who are clinically followed over a 2‐year observation period combined with regular microbiota sampling. Longitudinal studies and studies that fulfil the ‘commensal Koch's postulates’ are needed to put the gut microbiota dysbiosis connections with human health and disease on a firm scientific basis. Such claims might sound like scientific rigourism since it implicates many years of further research. However, we should be aware of what is needed for a proof in the dysbiosis/microbiome field, so as not to be tempted into premature conclusions and thus creating unrealistic hopes for translation of microbiome research into amelioration of human health.

Clinical intervention trials are finally the critical test for the practical value of microbiome research. The success of faecal transplantation in *C. difficile* infection is a sign of hope (Tariq *et al*., 2019), but we need to decipher how it works mechanistically, both to achieve an industrial product and to understand why faecal transplantation works less well in other gastroenterology diseases (Imdad *et al*., 2018).

## Conflict of interest

None declared.
